# Structural Basis of a Novel Heme Binding Bacterial One-Component Switch

**DOI:** 10.64898/2026.03.15.711900

**Published:** 2026-03-15

**Authors:** James J. Siclari, Malvin Forson, Cullen Roeder, Eta A. Isiorho, Denize C. Favaro, Rinat R. Abzalimov, Stephen S. Gisselbrecht, Alec H. Follmer, Martha L. Bulyk, Kevin H. Gardner

**Affiliations:** 1 Structural Biology Initiative, CUNY Advanced Science Research Center, New York, NY 10031; 2 Ph.D. Program in Biology, The Graduate Center – City University of New York, New York, NY 10016; 3 Ph.D. Program in Biochemistry, The Graduate Center – City University of New York, New York, NY 10016; 4 Division of Genetics, Department of Medicine, Brigham and Women’s Hospital and Harvard Medical School, Boston, MA 02115; 5 Department of Chemistry, University of California -Davis, Davis, CA 95616, USA; 6 Department of Pathology, Brigham and Women’s Hospital and Harvard Medical School, Boston, MA 02115; 7 Ph.D. Programs in Biochemistry, Biology, and Chemistry, The Graduate Center – City University of New York, New York, NY 10016; 8 Department of Chemistry and Biochemistry, City College of New York, New York, NY 10031

**Keywords:** Per-ARNT-Sim domains, Bacterial Signaling, Heme Binding Proteins, One-Component Systems

## Abstract

One-component systems (OCSs) integrate sensory and effector functions within a single protein, enabling rapid gene expression changes in response to environmental cues. Here, we characterized a novel putative OCS protein, FG214, from *Fimbriimonas ginsengisoli*, which drew our attention as a potential redox or O_2_-regulated helix-turn-helix (HTH)-Per-ARNT-Sim (PAS) transcription factor. Data supporting this included our observation of the FG214 PAS domain binding a hexacoordinate heme b in oxidized conditions and undergoing a slate of redox and ligand-dependent conformational changes, transitioning from a monomer to a homodimer. Spectroscopic and structural data revealed that oxidation stabilizes the likely HTH-PAS intramolecular domain interface, while reduction of the heme iron dissociates the HTH, freeing previously-sequestered homodimerization surfaces. Similar effects were seen by addition of a small molecule ferric heme ligand, as directly visualized with a 1.47 Å crystal structure of an imidazole-bound truncated construct. Using *in vitro* DNA-binding assays, we identified an artificial promoter sequence and demonstrated ligand-enhanced protein-DNA binding. Finally, we performed proof of concept experiments exploring the ability of FG214 to homodimerize *in vivo*, setting the stage for a redox or gas sensitive biosensor. Together, these findings define FG214 as a novel heme-binding PAS DNA binding protein and potential transcription factor, complementing known heme-PAS two-component signaling switches.

## Introduction

Bacteria rely on an array of sensory and signal transduction proteins to detect and respond to environmental fluctuations. Many such responses are mediated by one-component systems (OCS), in which the sensory and effector domains reside within a single polypeptide(1). Among these proteins, Per-ARNT-SIM (PAS) domains are often found playing sensory roles given their remarkable adaptability, binding diverse small molecules to allosterically regulate partner domains differentially (1–4).

A subset of bacterial PAS domains coordinate heme prosthetic groups, enabling direct sensing of gases, redox potential, and metabolic state(5). Much of our understanding of this regulation stems from one such type of system, the *Bradyrhizobia* FixL histidine kinase-FixJ response regulator pair, where changes in O_2_ occupancy at the distal position alters net kinase activity of FixL, along with subsequent FixJ phosphorylation and transcriptional activation. However, questions remain open about both FixL signaling – particularly how signals are transduced from the hemes to activate this enzyme – and more broadly about their applicability to other heme-PAS proteins(6–9).

Beyond their physiological roles, PAS-containing OCS proteins offer powerful platforms for biosensing and synthetic regulation due to their compact architecture and modular signaling logic. The blue-light responsive activator EL222, for example, undergoes a conformational switch upon illumination that releases its helix-turn-helix (HTH) effector, activating transcription(10–13). Previous work from our lab and others have engineered this system for uses in prokaryotic and eukaryotic model systems (14–20). Similar sensory logic could, in principle, be harnessed for small molecule or redox driven systems.

Here we describe FG214, a HTH-PAS transcription factor from *Fimbriimonas ginsengisoli*(21), that binds a heme b within its PAS domain using two protein histidine sidechains to an oxidized Fe(III) as purified *in vitro*. We show that redox changes or exogenous imidazole induce structural rearrangements which convert FG214 from a monomer into a homodimer, enabling DNA binding. Our work identifies a novel one-component heme binding PAS protein and establishes a mechanistic framework for redox-regulated “effector release” while also providing proof of concept experiments for its utility as an *in vivo* biosensor. These findings expand the functional landscape of PAS domains and suggest new strategies for engineering redox-sensitive systems.

## Results

### Redox-dependent global conformational changes

**Figure 1:**
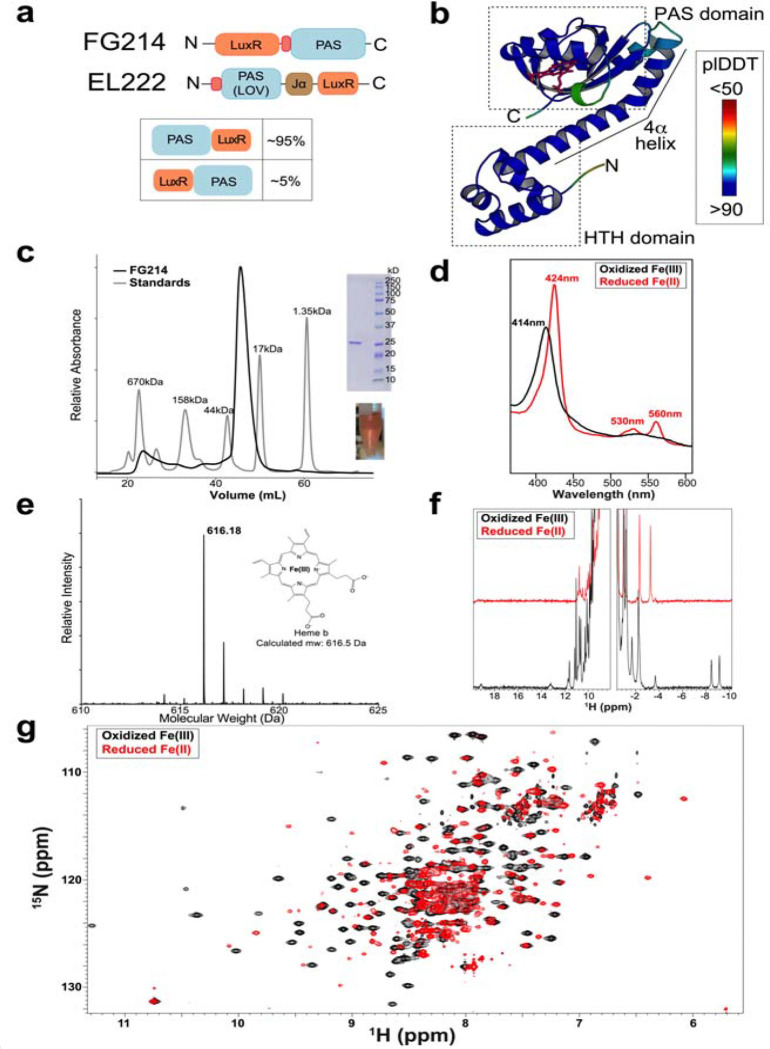
FG214 is a redox-sensitive heme-binding protein. **a)** Domain architecture of FG214 and another PAS-containing transcription factor, EL222. **b)** AlphaFold3 model of FG214, highlighting locations of HTH and PAS domains, as well as the connecting 4α helix. **c)** Superdex S200 SEC chromatogram of FG214 compared to standards of known molecular weight. (inset) SDS-PAGE gel verifying expected MW (24 kDa predicted from sequence) and image of purified protein. **d)** UV-visible absorbance spectra of oxidized (black) and reduced (red) FG214. **e)** Electrospray ionization LC-MS analysis confirming the presence of heme b within FG214 isolated from *E. coli*. **f)** ^1^H NMR spectra of oxidized (black) and reduced (red) FG214. **g)** ^15^N/^1^H TROSY spectra of oxidized (black) and reduced (red) FG214.

FG214 was identified in a bioinformatics screen for PAS domain-containing transcription factors with potential chemosensory triggers(22). These analyses predicted an N-terminal LuxR-type DNA-binding tetrahelical helix-turn-helix (HTH) DNA-binding domain and a C-terminal PAS domain, connected by a predicted helical linker. While these two types of domains are commonly found together (over 6000 occurrences in SMART(23) as of Feb 2026), the vast majority of these have a domain architecture with an N-terminal PAS sensor and C-terminal LuxR HTH DNA-binding domain, as previously seen in EL222(10) (Fig. 1a,b). Following overexpression and purification from *E. coli*, FG214 was visibly red, consistent with metal binding. Subsequent UV-visible absorbance spectroscopy and LC-MS revealed a heme b ligand(24) (Fig. 1c,d,e), and redox titrations demonstrated reversible spectral changes consistent with a redox-active heme(25–27) (Fig. 1d).

Reduction of the heme iron induced widespread structural changes detected by ^1^H and ^15^N/^1^H TROSY NMR (Fig. 1f,g). In the oxidized state, the spectra exhibited well-dispersed resonances of uniform intensity, suggesting a folded protein. We observed ^1^H peaks of oxidized FG214 distinctively upfield of 0 ppm, likely arising from protons interacting with a low-spin ferric Fe(III) state in the heme(28). These peaks were greatly perturbed by reduction, typical of a paramagnetic Fe(III) to diamagnetic Fe(II) spin system transition(29).

More globally, reduction caused extensive chemical shift perturbations and peak broadening in FG214 ^15^N/^1^H TROSY spectra, suggestive of a large-scale protein conformational change. While we lack the chemical shift assignments needed to fully establish the nature of this event using solely solution NMR, we can establish two key features by comparing spectra acquired from a series of FG214 truncations (1–214, 73–214 [Δ72], 87–214 [Δ86]) (Fig. S1). Several peaks in this series that are present in all three spectra – and hence must arise from residues in the PAS domain or an N-terminal 25 residue section – show a progressive linear change in chemical shift, strongly suggesting that the truncations alter an equilibrium. Coupled with AlphaFold3 models of the variants, this pattern was highly reminiscent of the light-triggered helix release equilibrium seen in the photosensitive *As*LOV2 system with an FMN-bound PAS domain(30, 31). These same peaks exhibited reduction-triggered chemical shifts as well, strongly suggesting that a heme-based mechanism also alters this equilibrium *in vitro* (Fig. S1). Taken together, our solution NMR data indicate substantial redox-triggered changes in FG214 structure suggestive of a triggered release of N-terminal regions before the PAS domain.

### Reduction promotes release of the HTH domain

**Figure 2:**
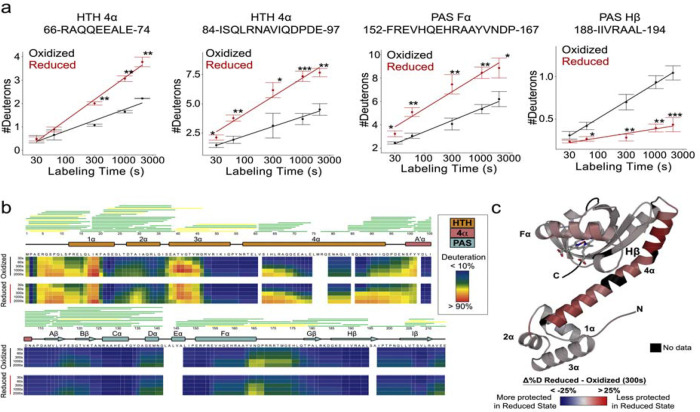
HDX-MS indicates that FG214 employs an effector-release mediated mode of activation. **a)** Peptide deuteration uptake plots of selected regions of FG214 under oxidized (black) or reduced (red) conditions. **b)** Heat map showing percent change in deuteration over time between oxidized and reduced states. Secondary structure diagram shows HTH domain in orange, A α in pink, and PAS domain in cyan. **c)** Heat map of reduction-triggered changes in HDX protection as data mapped onto FG214 AlphaFold3 model. Red color indicates less protection in reduced form; blue indicates more protection in reduced form; black indicates no data due to gaps in peptide coverage. Asterisks denote statistical significance derived from a two-tailed Welch’s t-test.

Hydrogen-deuterium exchange mass spectrometry (HDX-MS) was used to elucidate how heme reduction shifts the conformational landscape of FG214. HDX-MS protection patterns of both the oxidized and reduced form of the protein are generally consistent with AlphaFold3 predictions, including placements of all 2° structures in both domains. The predicted long 4α helix is also visible in these protection patterns, but notably, it shows a marked increase in deuteration levels upon reduction (Fig. 2a,b,c). This interdomain 4α helix appears to make direct contact with the FG214 PAS domain in the oxidized monomer AlphaFold3 model. Our experimental data clearly suggests reduction destabilizes the 4α helix from the PAS core, consistent with an allosteric release of the effector domain. This is a mechanism seen multiple times previously in prokaryotic and eukaryotic signaling proteins(31–34). We also see reduction influenced changes in the PAS domain around the known ligand binding regions, mainly in the Eα helix and Hβ strand, consistent with a redox-triggered shift in the geometry or volume of the heme binding pocket. These features suggest that the oxidized state adopts a stable monomeric “off” conformation, whereas reduction destabilizes the fold.

### Heme coordination chemistry is linked to FG214 dimerization

**Figure 3:**
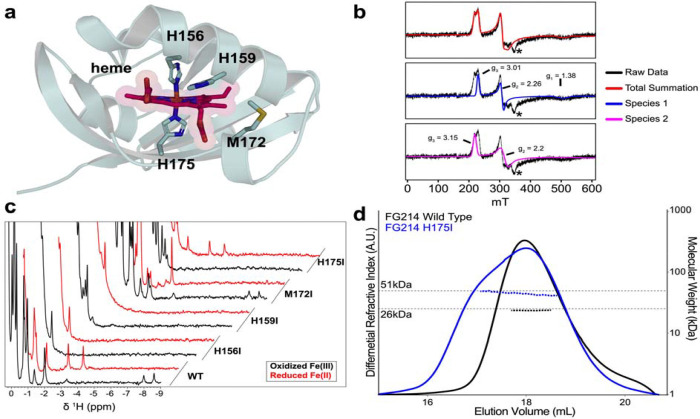
FG214 binds heme with two histidine residues in the oxidized state. **a)** AlphaFold3 model of full length FG214 heme binding pocket. **b)** X-band EPR spectra of oxidized FG214 showing two different low spin ferric states. Asterisk indicates a small background Cu signal. **c)** ^1^H NMR spectra of oxidized (black) and reduced (red) wild type (bottom, reproduced from Figure 1f) and point mutants of potential liganding residues. **d)** SEC-MALS using a Superdex 200 column of WT (black) and H175I (blue) FG214 proteins.

The iron in heme b is typically coordinated by a combination of histidine and/or methionine residues in the oxidized state(35). Identifying the coordinating residues within FG214 allows for targeted mutagenesis to probe structure-function relationships and potentially shift the equilibrium between inactive and active conformations. Guided by AlphaFold3 structural predictions (Fig. 3a), we identified four candidate residues (His 156, His 159, Met 172, His 175) and sought to experimentally determine their roles in heme coordination.

As an initial examination, we acquired X-band electron paramagnetic resonance (EPR) spectra of oxidized FG214, revealing two rhombic S=1/2 populations (Fig 3b). Both species had g-values supporting the assignment of 6-coordinate low spin-ferric heme, sometimes referred to as highly anisotropic/axial low-spin (HALS) systems(36). The first, less rhombic species exhibited g-values (3.01, 2.26, 1.38) similar to many other HALS proteins where the heme cofactors are bound by two histidine residues arranged in an antiparallel geometry (Fig. 3b), consistent with our ^1^H NMR spectra displaying upfield-shifted resonances (Fig 1f). The second species is more rhombic and only two g-values are observed in this magnetic field range (g-values: 3.15, 2.2). While we cannot rule out that this second species arises from a second orientation of the bis-His coordination, it is also possible that this more rhombic population indicates a potential His-Met coordination in the oxidized state with similar g-values observed for these systems(36).

To further test this model, we generated a series of single point mutations targeting the four histidine and methionine residues predicted in the binding pocket and substituting each with isoleucine to maintain hydrophobicity and approximate sidechain volume. We used UV-visible absorbance spectroscopy to compare relative heme loading across mutants, using samples with matched protein concentrations (as judged by A_280_ values) and, focusing on changes in the Q- and Soret band regions. While M172I exhibited near-wildtype ability to bind heme as judged by the Q- and Soret band peak intensities, the other three mutants were impaired. This was most evident with the H156I mutant, particularly with the near absence of a Soret band in the reduced state (Fig. S2, Table S1). The H159I mutant also exhibited a distinct spectral signature, including a new absorbance maximum at 662 nm and an altered color in solution, reflecting perturbed coordination geometry (Fig. S2, Table S1). The persistence of partial binding among all mutants suggests compensatory heme coordination, underscoring some degree of pliability in the FG214 heme binding site.

^1^H NMR spectra and size exclusion chromatography of the mutated proteins further revealed structural changes caused by these substitutions. Focusing first on the NMR signals upfield of 0 ppm, H156I and H159I displayed substantial peak losses in this region, consistent with global destabilization of the protein (Fig. 3c). M172I and H175I were subtler in their changes, but notably with oxidation-specific effects, with M172I being most strongly perturbed in the reduced form and H175I in the oxidized state. Coupled with UV-visible absorbance data and the AlphaFold3 prediction, we assign His 156 (proximal) and His 175 (distal) as the heme-coordinating residues, with some potential role for Met 172 as an alternative distal participant.

To explore the roles of these heme-coordinating residues in the FG214 quaternary structure, we used size-exclusion chromatography with inline light scattering (SEC-MALS) to characterize changes in solution shape and mass. While the lack of heme binding by H156I precluded us from acquiring meaningful SEC-MALS data on this variant, we obtained data clearly showing that wildtype FG214 is monomeric in solution and H175I is markedly shifted towards a dimeric species (Fig. 3d). Taken together, our solution data clearly support a model where changes near the heme site affect both an intramolecular HTH-PAS interaction predicted in the oxidized state, leading to protein dimerization.

### Crystal structure of the activated dimer

Guided by truncation analysis (Fig. S1), we successfully crystallized an imidazole-bound construct lacking the first N terminal 72 residues (Δ72), encompassing part of the HTH 4α helix through the PAS domain. In solution, this construct shares many features in common with the full-length protein, including being monomeric by SEC-MALS when oxidized, having nearly identical UV-visible absorbance characteristics, and similar ^1^H NMR shifts – with a notable shift in the location of heme vinyl chemical shifts near −8 ppm, showing that changes outside the PAS domain impact the environment near the heme (Fig. S3). During crystallization trials for FG214, we observed that addition of imidazole was required to obtain crystals, which we attribute to imidazole’s well-characterized ability to ligand ferric heme proteins. In doing so, it is known to bind the distal coordination site of heme, outcompeting native protein sidechain interactions, reproducing the electrostatic properties of Fe(II)-O_2_ complexes(37, 38).

**Figure 4:**
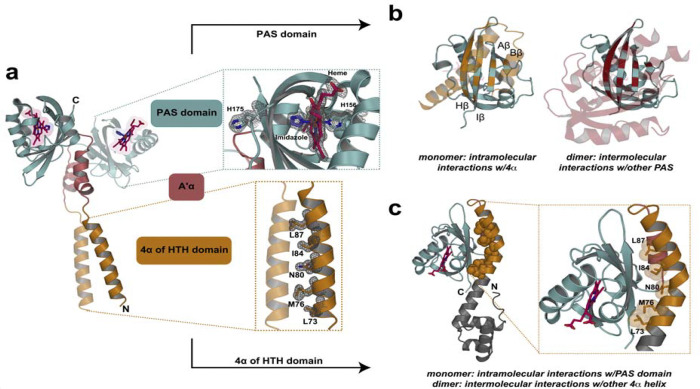
Crystal structure of an N-terminally truncated FG214 protein reveals imidazole driven dimerization. **a)** 1.47 Å crystal structure of FG214(Δ72). PDBID:10JX. Mesh shows electron density. Insets show heme binding cavity and hydrophobic interface of HTH 4α helix. **b)** FG214 PAS domain highlighting residues 4 Å away from the 4α helix in the AF3 monomer model (left) and 4 Å from the other PAS domain in the dimeric crystal structure (right). **c)** AlphaFold3 model of oxidized full length FG214 highlighting the locations of the 4α residues involved in the dimeric structure of Figure 4a.

From these imidazole-bound FG214(Δ72) crystals, we obtained a 1.47 Å structure of an imidazole-bound homodimer (Fig. 4a), mediated by both HTH-HTH and PAS-PAS interactions. Within the PAS domain, H156 serves as the proximal heme-coordinating residue, while an imidazole molecule occupies the distal site, fully corroborating our spectroscopic and mutagenesis data. Meanwhile, the H175 sidechain, which we expect to normally serve as the distal ligand, was displaced into an unstructured loop that meets the other monomer at the dimerization interface. Of note, the nearby M172 sidechain is similarly outside of the PAS domain in one of the two chains but is not resolved in the other. These structural changes clearly implicate linkage between heme coordination and protein oligomerization state together.

Examining the FG214 dimer, we saw potential interactions between the two chains mediated by both PAS/PAS and 4α/4α contacts. At the PAS domains, these were chiefly involving A α helix/β-sheet and β-sheet/β-sheet interactions; of note, the β-sheet residues are also predicted by AlphaFold3 to make an intramolecular monomeric interface with 4α (Fig. 4b). At the HTH domains, hydrophobic sidechains of the two 4α helices interact to form the dimer; reminiscent of coiled-coil interfaces of transcription factors(39–41). And again, the 4α residues at the dimer interface are predicted to play an integral role in stabilizing the monomeric form (Fig. 4c).

Soaking these same crystals with sodium dithionite (DT) yielded datasets leading to a 1.67 Å structure of FG214(Δ72), revealing a homodimer with nearly identical 4° structural arrangements as previously seen. However, there are distinct changes at the heme – which has a His 156-Met 172 iron coordination pair and His 175 on an extended loop pointing into solution (Fig. S4).This raises the potential that alternative His-His and His-Met heme coordination pairs – as suggested by our EPR and mutagenesis results in the full length protein (Fig. 3) – may exist and be biased in different crystal forms of FG214 variants. Indeed, AlphaFold3 modeling suggests that removal of the HTH domain favors His-Met coordination in the monomer (Fig. S5), perhaps due to altered states of the outer β-sheets within the PAS core due to the shortened 4α helix. Regardless, these structural data strongly implicate changes at the heme site being amplified by the surrounding protein to affect the monomer:dimer equilibrium of FG214, as is commonly seen as a regulatory mechanism in other proteins.

### Identification of an artificial DNA binding site

**Figure 5:**
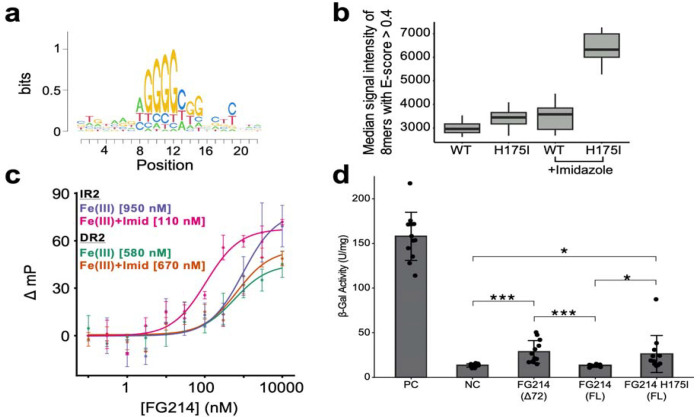
FG214 binds a GC-rich palindromic promoter region enhanced by imidazole in the ferric state. **a)** DNA half-site for FG214 binding identified by universal protein-binding microarray (PBM). **b)** Median intensity of specifically-bound 8-mers in PBM for FG214 WT and H175I in the absence and presence of 500 mM imidazole. **c)** Fluorescence polarization measurements of 1 nM FAM-labeled DR2 or IR2 sequences with increasing concentrations of FG214 in the absence and presence of 10 mM imidazole. All individual measurements were collected in triplicate; points shown are average ± 1 standard deviation. **d)** β-galactosidase activity quantified following bacterial two hybrid (BTH) analysis of split adenylyl cyclase vectors (NC) or fused to leucine zippers (PC), FG214 (Δ72), FG214 full-length, or FG214 full-length H175I. Statistical significance derived from a two-sample t-test, with *** = 0.0001 < p < 0.001 and * = 0.01 < p < 0.05. Individual datapoints are shown, with top bar drawn at mean value and error bars showing ± one standard deviation from the mean.

To explore the functional importance of these changes, we aimed to identify a suitable artificial DNA sequence for FG214 binding, both to enable tests of our hypothesis of imidazole-driven activation and inform downstream functional assays and future bioengineering. To do so, we used universal protein binding microarrays (PBMs) to screen FG214 for *in vitro* sequence-specific DNA binding activity against all possible 8 bp sequences(42, 43). Analysis of PBM-derived scores from these experiments of these data yielded a DNA binding specificity motif for FG214 (Fig. 5a), with a notable bias towards a purine-rich strand on one side of an 8 bp sequence.

Given the role of the distal His 175 in maintaining the FG214 monomer, we also tested whether ligand-induced displacement of this sidechain in a wild-type background could promote DNA binding. Analysis of PBM fluorescence intensities across the array revealed enhanced binding by the H175I mutant relative to wild-type FG214, with further increases for both constructs in the presence of 500 mM imidazole (Fig. 5b). These observations suggest that imidazole-induced displacement of His 175 facilitates domain rearrangements sufficient for dimer-mediated DNA binding, thereby partially activating FG214 under ferric conditions. These findings align with prior studies showing that exogenous imidazole can act as a functional mimic for *in vivo* heme ligands(44–46). In this context, imidazole binding appears to trigger an active conformation of FG214 without the need for heme reduction.

To develop an optimal high-affinity DNA binding site for *in vitro* assays, we constructed a series of DNA sequences containing direct or inverted repeats of the PBM-identified motif (GGGGCGGGG), assuming that this sequence is a likely half-site for binding a FG214 dimer. We generated 14 FAM labeled binding substrates with varying half-site orientation and spacer length from 1–7 bp (Fig. S6). Each DNA construct was labeled with FAM (fluorescein derivative) on the 5 end to enable quantitative binding analysis via fluorescence polarization/anisotropy. Oligos containing direct repeats of the motif bound FG214 weakly and non-specifically, and without any dependence on mutation or imidazole concentration (Fig. S6). In contrast, oligos with inverted repeats of the motif displayed stronger binding, particularly for the H175I variant, and often with imidazole dependence (Fig. S6). From these experiments, a particularly clear exemplar of FG214 DNA binding characteristics are evident from oligos with a 2 bp central spacer: Inverted copies of this motif (IR2; Table S3) exhibited the most pronounced imidazole-dependent binding response (without imidazole: K_d_ ≈ 950 nM; with 10 mM imidazole, affinity increased by approximately an order of magnitude to an apparent K_d_ ≈ 100 nM) (Fig. 5c). In contrast, direct repeats around the same 2 bp spacer (DR2; Table S3) showed moderate binding of K_d_ ≈ 600 nM with no imidazole dependence.

### *In Vivo* Validation of FG214 homodimerization

To explore the potential for FG214 to adopt the homodimeric state that appears to be required for DNA binding, we employed a bacterial two hybrid (BTH) assay to probe FG214 homodimerization in *E. coli.* By fusing FG214 (full length, mutant, or the Δ72 truncation) to fragments of a split adenylyl cyclase we can analyze FG214 homodimerization following co-transforming into *E. coli* lacking its native adenylyl cyclase(47, 48). Here, complementation of the split cyclase will only occur with sufficient FG214 homodimerization, facilitating cyclic AMP (cAMP) production from ATP and activating CAP-dependent expression of a β-galactosidase which can be easily assayed on plates or in solution as a proxy for dimerization. Employing this BTH assay by quantitatively measuring β-galactosidase activity in cell lysates in the presence of orthonitrophenyl-β-D-galactopyranoside (ONPG), we saw significant increases in β-galactosidase activity in FG214(Δ72) and the H175I full length fusions compared to the negative control (NC), confirming of FG214 homodimerization in *E. coli* grown under standard aerobic conditions (Fig. 5d). No significant homodimerization was seen for full-length FG214, indicating that these growth conditions are insufficient on their own to trigger homodimerization. Taken together, these cell-based data are all consistent with our *in vitro* biochemical and structural results.

## Discussion

**Figure 6:**
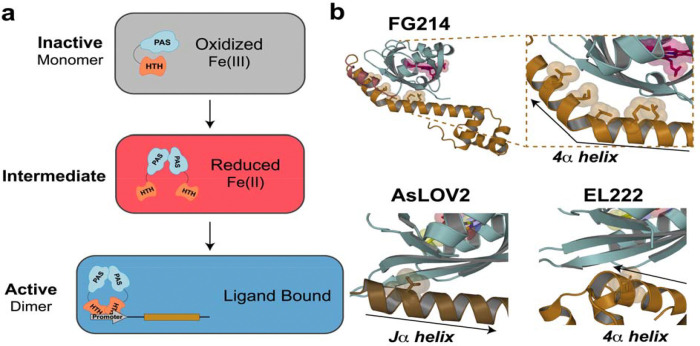
Proposed signaling model for FG214, with a signal-induced monomer to dimer transition activating DNA binding, like other PAS domain sensors. **a)** Proposed schematic of FG214 activation. **b)** EL222 and AsLOV2, PAS domain containing proteins that rely on interactions between auxiliary helices and β-sheet surfaces to maintain inactive states. Note that auxiliary helices can originate either N- or C-terminal of the PAS domain and run in either direction across PAS β-sheet surface.

Our work provides structural and mechanistic insight into a heme-binding PAS-HTH one-component DNA-binding protein, FG214. By characterizing the monomeric inactive oxidized state, the intermediate reduced state, and the active DNA-bound homodimer triggered by imidazole, we define three likely signaling states of the protein (Fig. 6). Together, these data support a model in which FG214 employs an effector-release activation mechanism to sense and respond to environmental gases, a framework previously observed in other PAS-containing signaling systems. In addition to expanding our knowledge of bacterial signaling proteins, we have laid the foundation for the development of FG214-based transcriptional reporter systems.

### Effector-release mechanism

Structural and dynamic analyses of the oxidized FG214 monomer validate the AlphaFold prediction for the HTH 4α helix to form key contacts with the PAS domain β-sheet, particularly via HDX-MS analysis showing this helix to be well ordered. This conformation masks HTH and PAS surfaces required for dimerization, as demonstrated by our solution measurements. Upon Fe(III) reduction or ligand binding, however, this helix is markedly destabilized, suggesting that helix unwinding facilitates dimerization and activation. Indeed, our crystal structures of reduced and imidazole-bound FG214(Δ78) confirm that the newly-freed PAS β-sheet and HTH 4α helix contribute substantial homodimer interfaces, supporting a model in which allosteric changes initiated at the heme cofactor reorganize nearby FG214 sheet/helix interactions as a key trigger to transition from a monomeric “off” state to a dimeric DNA-binding “on” state.

This activation mechanism parallels those used by certain other PAS sensors, including the light-activated AsLOV2 and EL222 from the LOV subfamily(10, 12, 30) (Fig. 6). In those cases, allosteric signals triggered by illumination or redox changes at an internal flavin cofactor relay across the LOV β-sheet, displacing the Jα helix (AsLOV2 (30, 31)) or the 4α helix (EL222 (10)) as part of the activation process. We underscore that the helices involved in these stimuli-dependent binding/unbinding equilibria bind to equivalent spots on the PAS β-sheet, despite coming from very different locations in the primary sequence compared to the PAS domain itself – from immediately C-terminal (AsLOV2 Jα) to 70–80 residues C-terminal (EL222 4α) to 20–40 residues *N-terminal* (FG214 4α) – and binding in different orientations (Fig. 6b). This suggests evolutionary conservation of a common signaling mechanism despite marked differences as substantial as the order of domains(49, 50). Upon freeing the 4α helix, the FG214 β-sheet becomes accessible for homodimerization, utilizing the same residues just liberated to do so (Fig. 4b,c). This is very reminiscent of the proposed signaling mechanism for EL222(10, 12, 13), despite differences in the controlling cofactor (heme b vs. FMN) and domain orientation (HTH-PAS vs. PAS-HTH).

### DNA binding specificity and implications for dimerization

Our DNA-binding data across constructs with varying spacer lengths provide key information into the monomer-dimer equilibrium of FG214 as well as functional data of the active state. We interpret the binding to direct-repeat sequences – relatively weak, relatively low change in fluorescence polarization, and chiefly unaffected by changes in spacer, addition of imidazole or the H175I mutation – likely reflects low-affinity recruitment of monomeric FG214 to individual half-sites. In contrast, inverted repeats displayed higher affinity, more stimulus dependence, and more variation by spacer length. Coupled with the higher ΔmP for the saturated inverted repeats when compared to the direct, these data are consistent with engagement by an activated dimeric form of FG214. Given that GC rich regions are implicated in transcriptional control of oxidative stress genes(51–53), which appear throughout the native *Fimbriimonas ginsengisoli* genomes, we speculate that FG214 may have an *in vivo* role controlling the expression of these genes, but this remains to be experimentally validated. While such cellular experiments are outside the scope of this work, we believe that the correlation we see between *in vitro* and *E. coli* dimerization of FG214 truncation or the H175I mutation – a step required for DNA binding – provides both useful reagents and frameworks for those studies.

### Comparison with other heme-binding PAS sensors

Heme-binding PAS domains have been previously reported in other proteins, but never in an OCS context. Of these, several gas-sensing bacterial PAS proteins have been best studied, including FixL, Aer2, and EcDos/DosP(54–57). Other proteins, like the FlrB histidine kinase, use the PAS fold to detect levels of heme directly as opposed to using heme as a prosthetic group to sense secondary ligands, as bacteria often sequester heme from symbionts or host organisms as an iron source(58–60).

Among known heme-binding PAS proteins, the oxygen-sensing kinase FixL remains the best characterized and is often regarded as the canonical model. In *Bradyrhizobium japonicum*, FixL forms part of a two-component system in which the heme-binding PAS domain modulates kinase activity in response to oxygen levels. In its oxidized state, FixL binds heme in a pentacoordinate geometry through a single proximal histidine(46, 61, 62). Researchers also used ligands like imidazole or cyanide to mimic O_2_ binding under oxidized conditions *in vitro*(54, 63). Such binding events reshape a preformed PAS homodimer within FixL(64), leading to conformational changes within a coiled-coil linker to a downstream histidine kinase, controlling enzymatic activity as it does.

In contrast, FG214 coordinates heme via a chiefly bis-histidine ligation in the ferric state and undergoes distal coordination rearrangement upon activation. Subsequently triggered protein conformational changes are also distinctly different, as FG214 uses heme sensing to control a monomer:dimer equilibrium for activation. These different coordination chemistries and signaling mechanism illustrate how PAS domains have evolved diverse strategies to couple similar environmental cues and relay them into downstream signaling. The comparison underscores the remarkable modularity and adaptability of PAS domains across species and regulatory contexts.

### Implications for redox signaling and synthetic applications

By uncovering the molecular basis of heme-based sensing in FG214, we have revealed how structural modularity of PAS domains within one-component systems enables evolutionary diversification of signaling logic to respond to different signals with different domain orientations while retaining some mechanistic similarity with effector release. We suggest that FG214 thus represents a new prototype for heme-regulated transcriptional switches, expanding the known repertoire of OCS PAS signaling mechanisms beyond light and ligand-sensing systems, and laying the foundation for novel engineered redox- or gas-sensitive proteins. Such systems could be valuable by both informing aspects of natural biological signaling and in a range of biotech or environmental applications where control of biological processes by redox or heme-binding ligands may be useful.

## Materials and Methods

Proteins were expressed in *E. coli* BL21(DE3) cells and purified through nickel-affinity chromatography and size exclusion chromatography. UV-visible absorbance spectra were collected on a Varian Cary 60 spectrophotometer. NMR data were collected using a Bruker Avance III HD 800 MHz (18.8 T) spectrometer with a 5 mm TCI CryoProbe at 298K. Crystallographic data were collected at National Synchrotron Light Source II (NSLS-II) light at Brookhaven National Laboratory on beamline 19-ID (NYX) and processed using the autoPROC toolbox(65). DNA binding was assessed by fluorescence polarization using FAM-labeled dsDNA collected with a Spectramax I3 equipped with a fluorescence polarization cartridge (Molecular Devices) using a sequence identified from a protein binding microarray(42). Detailed descriptions of all methods are available in *SI Appendix*.

## Supplementary Material

Supplement 1

## Data Availability

The X-ray structure coordinates for truncated FG214 with and without Na_2_S_2_O_4_ are available from the Protein Data Bank under accession codes 10JY and 10JX respectively. PBM data are deposited in the NIH Gene Expression Omnibus (GEO) under accession GSE319048. All other data are available upon request to K.H.G. on behalf of the authors.

## Significance Statement

Studying sensory protein structure-function relationships is paramount to fully understanding cellular adaptation to environmental cues. Here, we have discovered a heme-regulated DNA binding switch, FG214, that undergoes a monomeric to dimeric transition upon redox-triggered changes. The nature of this transition is reminiscent of other proteins with similar domain architecture, although those have been observed to instead sense changes in the presence of other distinct stimuli. Here we not only describe the activation mechanisms of FG214, but also provide support for its ability to be used as a regulatory gene expression tool.
