# Supplementary material for: Structural Basis of a Novel Heme Binding Bacterial One-Component Switch
Source: bioRxiv. 2026 Mar 15:2026.03.15.711900. Preprint. [Version 1] doi: 10.64898/2026.03.15.711900 (PMC13060918; doi:10.64898/2026.03.15.711900)
Supplement: Supplement 1 [file media-1.docx]

**Structural Basis of a Novel Heme Binding Bacterial One-Component Switch**

Supporting Information

James J. Siclari^1,2^, Malvin Forson^1,3^, Cullen Roeder^1,3^, Eta A. Isiorho^1^, Denize C. Favaro^1^, Rinat R. Abzalimov^1^, Stephen S. Gisselbrecht^4^, Alec H. Follmer^5^, Martha L. Bulyk^4,6^, Kevin H. Gardner^1,7,8,^*

*: direct correspondence to [kgardner@gc.cuny.edu](mailto:kgardner@gc.cuny.edu)

^1^: Structural Biology Initiative, CUNY Advanced Science Research Center, New York, NY 10031

^2^: Ph.D. Program in Biology, The Graduate Center – City University of New York, New York, NY 10016

^3^: Ph.D. Program in Biochemistry, The Graduate Center – City University of New York, New York, NY 10016

^4^: Division of Genetics, Department of Medicine, Brigham and Women’s Hospital and Harvard Medical School, Boston, MA 02115

^5^: Department of Chemistry, University of California -Davis, Davis, CA 95616, USA

^6^: Department of Pathology, Brigham and Women’s Hospital and Harvard Medical School, Boston, MA 02115

^7^: Ph.D. Programs in Biochemistry, Biology, and Chemistry, The Graduate Center – City University of New York, New York, NY 10016

^8^: Department of Chemistry and Biochemistry, City College of New York, New York, NY 10031

Contents:

- Supporting Methodology
- Supporting Figures S1-S6
- Supporting Tables S1 & S2
- Supporting References

**Supporting Methodology**

Cloning, Protein Expression and Purification

DNA encoding the FG214 sequence (GenBank: CP007139 Region: 3534516 – 35335172, UNIPROT ID: A0A068NTE8) was ordered from Twist Biosciences and cloned in a pHisGβ1-parallel expression vector (1). FG214 was recombinantly expressed with a His6-Gβ1 tag in BL21(DE3) *E. coli* (Stratagene) in M9 minimal media plus 1 mM 5-aminolevulinic acid at 16°C overnight. Expression was induced with 1 mM isopropyl β-D-1-thiogalactopyranoside. Cells were pelleted and resuspended in Buffer A (50 mM Tris-HCl pH 8.0, 500 mM NaCl). The cells were lysed by sonification, and the lysate was cleared by centrifuging at 10,000 x g for 30 minutes at 4°C. The supernatant was collected and filtered through a 0.2 µm syringe filter. For affinity purification, the protein was loaded onto a 5 mL HisTrap (Cytiva) column preequilibrated with Buffer A. The column was washed with 10 column volumes (CVs) of Buffer A before eluting the protein with buffer B (50mM Tris-HCl pH 8.0, 500 mM NaCl, 500 mM imidazole). The eluent was diluted 1:10 with Buffer C (50 mM Tris-HCl, pH 8.0) and the His_6_-Gβ1 tag was cleaved by adding 1 mg His_6_-TEV protease (2, 3) per 30 mg of fusion protein. After overnight cleavage, the sample was again applied to a preequilibrated 5 mL HisTrap column to remove tag and TEV protease. The flow through was then concentrated using a 30 kDa MWCO Amicon Ultra concentrator to a volume of 3 mL. The concentrated flowthrough was applied to a Superdex 200 10/300 (Cytiva) column preequilibrated with assay buffer (50 mM sodium phosphate pH 7.0 and 50 mM NaCl) unless otherwise noted.

UV-Visible Absorbance Spectroscopy

FG214 was concentrated to 15 µM in 100 mM sodium phosphate and 50 mM NaCl pH 7.0. Using a 1cm path length quartz cuvette, oxidized spectra were recorded at room temperature using a Varian Cary 60 from 250-600 nm. To reduce the sample, a stock solution of sodium dithionite was made in buffer that previously degassed for 1 hour and added to the sample to a final concentration of 10 mM.

Mass Spectrometry

To confirm the presence of heme B bound to the protein, we performed a fast SEC-MS experiment using a short SEC column on Dionex Ultimate 3000 LC-system coupled to a Bruker maXis-II ESI-QqTOF mass spectrometer. Briefly, 10 µL of a 10 µM FG214 stock solution was injected onto a Thermo Scientific NativePac OBE-1 SEC column to enable rapid exchange of non-volatile buffer components into an MS-compatible solvent (50 mM ammonium acetate as the mobile phase). The protein eluted within 2 minutes. We observed the holoprotein with the expected mass increase corresponding to bound heme B, as well as free heme B at m/z 616.18.

NMR Spectroscopy

^1^H solution-state nuclear magnetic resonance spectra were acquired at 100 µM on a Bruker Avance III HD 800 MHz spectrometer operating at a ^1^H frequency of 800.05 MHz and equipped with a 5mm TCI CryoProbe. All experiments were performed at 298K using the standard Bruker pulse sequence (zgesgp) with NS: 512 and TD: 4096. ^15^N/^1^H TROSY spectra were collected at 250 µM using the pulse sequence ^15^N/^1^H-WADE-TROSY (4) with NS:8 and TD:2048 (F2) x 180 (F1). Oxidized vs. reduced spectra were collected in a valved NMR tube (DWK Life Sciences) to ensure a sustained anaerobic environment. All NMR data were processed and analyzed using NMRFx Analyst (5, 6).

Hydrogen Deuterium Exchange Mass-Spectrometry

FG214 was concentrated to a final concentration of 40 µM in 100 mM sodium phosphate 50 mM sodium chloride pH 7.0 (with 10 mM sodium dithionite for reduced samples). The protein was diluted 1:14 in 100% D_2_O buffer with same components as the protein buffer, incubated for a defined periods of time at 4°C. The exchange reaction was quenched by adding and equal volume ice-cold quench buffer (3 M guanidine hydrochloride, 3% acetonitrile, and 0.8% formic acid, pH < 2.0), followed by on-line peptic digestion using a Waters Enzymate BEH Pepsin Column, peptide desalting with Hypersil Gold C18 trap column (10 mm) and peptide separation on a Hypersil Gold C18 column (50 mm length × 1 mm diameter, 1.9 μm particle size, Thermo Fisher Scientific). The time between quenching and on-line peptide elution into the mass spectrometer was 5–10 min, at 0–4 °C and pH 2.5, to minimize back-exchange, and peptides were analyzed on a Bruker maXis-II ESI-QqTOF high-resolution mass spectrometer. To minimize in-source back exchange, the instrument ion-source temperature was lowered to 180°C. Initial processing of raw mass spectrometry data files was done with Bruker Compass Data Analysis 6.1. Peptide identification based on accurate mass measurements, MS/MS data, and peptides list curation was performed using Bruker’s Biotools 3.2 and PIGEON (7). Sequence coverage was above 98% for each condition. Downstream data analysis was done using HDExaminer 3.3 (Trajan Scientific). HDExaminer was used for calculation of deuterium uptake profiles and associated standard deviations. Post-analysis quality checks of peptide level deuteration plots, secondary peptide identification and validation were performed using PFNet (7).

EPR Spectroscopy

Samples were prepared using 200 µL of 250 µM FG214 in assay buffer with 20% glycerol and frozen under liquid nitrogen. Continuous wave X-band EPR spectra were collected on a Bruker EMX spectrometer using Bruker Xenon software (version 1.2) at 10 K using liquid helium and an Oxford Instruments (Oxford, UK) ESR-900 cryogen flow cryostat equipped with an ITC-503 temperature controller. Spectra were simulated using EasySpin 5.2.36 with Matlab R2022 (8).

Size Exclusion Chromatography Multi-Angle Light Scattering

Samples and buffers were filtered with a 0.1 µm pore size filter before use. Samples were injected at onto a Superdex 200 GL 10/300 column pre-equilibrated with 100mM sodium phosphate and 50 mM NaCl pH 7.0. Following elution light scattering was measured by an inline miniDAWN TREOS and refractive index was measured using an Optilab rEX (Wyatt Technology). The entire process was carried out at room temperature. Data analysis and molecular weight calculations were performed using ASTRA V software (Wyatt Technology).

Crystallization, Data Processing and X-Ray Structure Determination

Commercially available Morpheus Fusion screen (Molecular Dimensions) was employed to find suitable crystallization conditions. The best diffracting crystals were from condition A4 which contained 0.1 M carboxylic acids mix (0.2 M sodium formate, 0.2 M ammonium acetate, 0.2 M sodium citate tribasic dihydrate, 0.2 M potassium sodium tartrate tetrahydrate and 0.2 M sodium oxamate), 0.12 M alcohols (0.2 M 1,6-hexanediol; 0.2 M 1-butanol; 0.2 M 1,2-propanediol; 0.2 M 2-propanol; 0.2 M 1,4-butanediol and 0.2 M 1,3-propanediol), 0.1 M buffer system 1 at pH 6.5 (0.4 M imidazole and 0.6 M MES monohydrate), and 37.5% precipitant mix 4 (25% v/v MPD, 25% PEG 1000, 25% w/v PEG 3350 w/v). Crystals used for phasing were from condition C7 from the same screen consisting of the following composition, 0.09 M halides (sodium fluoride, sodium bromide and sodium iodide), 0.12 M monosaccharide mix 1 (d-glucose, d-mannose, d-galactose, l-fructose, d-xylose, *N*-acetyl-d-glucosamine) 0.1 M buffer system 1 at pH 6.5 (0.4 M imidazole and 0.6 M MES) and 30% precipitant mix 1 (40% v/v PEG 500 MME and 20% w/v PEG 20000). Crystals were either cryoprotected immediately after opening the well or after a five-minute soak with 10 mM sodium dithionite (Na_2_S_2_O_4_) using LV CryOil (MiTeGen), looped, and flash-cooled in liquid N_2_ prior to data collection. Data were collected at National Synchrotron Light Source II (NSLS-II) light at Brookhaven National Laboratory on beamline 19-ID (NYX). All data sets (C7, A4, and A4 supplemented with Na_2_S_2_O_4_) were measured using 0.920187 Å wavelength and crystallized in space group P2_1_2_1_2_1_ (see Table S2). Data were processed using the autoPROC toolbox (9). Phasing of the A4 data set was achieved through a combination of molecular replacement (MR) with the AlphaFold3 model and single anomalous dispersion (SAD) method using the anomalous signal from the halides present in the crystallization conditions from C7 data set via AutoSol (SAD-MR). Phases for the reduced A4 data set were obtained via MR using the Na_2_S_2_O_4_ free structure. Several cycles of refinement were conducted using Coot (10, 11) and Phenix (12) and further optimization using the PDB_REDO server (Joosten et al. 2014). Final data collection, processing, and refinement parameters are provided in Table S2.

Universal Protein Binding Microarrays

Constructs of FG214 wild type and H175I were generated with C-terminal human influenza hemagglutinin (HA) and 6His tags and expressed as described above. Cells were pelleted and resuspended in Buffer A (50 mM Tris-HCl pH 8.0, 500 mM NaCl). The cells were lysed by sonification, and the lysate was cleared by centrifuging at 10,000 x g for 30 minutes at 4°C. The supernatant was collected and filtered through a 0.2 µm syringe filter. For affinity purification, the protein was loaded onto a 5 mL HisTrap (Cytiva) column preequilibrated with Buffer A. The column was washed with 10 column volumes of buffer A before eluting the protein with buffer B (50mM Tris-HCl pH 8.0, 500 mM NaCl, 500 mM imidazole). The eluent was applied to a Superdex 200 10/300 (Cytiva) column preequilibrated with assay buffer (50 mM sodium phosphate pH 7.0 and 50 mM NaCl).

Proteins were assayed simultaneously on separate chambers of one universal PBM (AMADID# 030236, Agilent Technologies, Inc.) as previously described (13) at a final concentration of 750 nM in EN buffer (138 mM potassium glutamate, pH 7.2, 12 mM sodium bicarbonate, 0.8 mM magnesium chloride) or PBS buffer, with or without the inclusion of 500 mM imidazole. PBM scans were analyzed using the Universal PBM Analysis Suite (14) to generate position weight matrices (PWM) and calculate E-scores and Median Signal Intensity for all 8-mers. The representative sequence logo in Figure 5a was generated from the wild-type FG214 PBS+imidazole PWM using the seqLogo package in R. Intensity distributions in Figure 5b show the Median Signal Intensities of 8mers with E-score greater than 0.4, representing the most preferred binding sites, for both alleles in EN.

Fluorescence Polarization

To create each construct used in the FP assay, FAM labeled ssDNA oligos were synthesized (Integrated DNA Technologies) and annealed to unlabeled reverse compliment ssDNA by applying a decreasing temperature gradient starting at 90°C. Assays were conducted with a constant 1 nM concentration of FAM-labeled dsDNA and increasing concentration of FG214 in the presence of 5 mM MgCl_2_. Imidazole was added to final concentration of 10 µM. Readings were recorded in all black 96-well plates and measured with a Spectramax I3 (Molecular Devices) equipped with a fluorescence polarization cartridge (Molecular Devices) with excitation/emission wavelengths at 485/525 nm. Data reduction was accomplished within the SoftMax software (Molecular Devices).

Bacterial Two-Hybrid Assays

FG214 constructs were cloned into the multiple cloning sites, attaching them N-terminal of the T18 and T25 fragments of the split adenylyl cyclase using the pUT18 and pKNT25 vectors from the BACTH System Kit (15). Plasmids were transformed into BTH101 cells (15) and grown on plates containing M63 medium, 1.5% agar, 50 µg/mL ampicillin, 50 µg/mL streptomycin, 50 µg/mL kanamycin, 120 µg/mL Bluo-gal, and 0.5mM IPTG at 30°C for 120 hours. Single colonies were picked and grown overnight in 3 mL of LB-Media with 50 µg/mL streptomycin, 50 µg/mL kanamycin, and 0.5mM IPTG at 30°C. Cultures were diluted 5x in M63 medium (15 mM (NH_4_)_2_SO_4_, 100 mM KH_2_PO_4_, 1.8 µM FeSO_4_, and 1mM MgSO_4_, 0.2% maltose) in 5 mL glass tubes. OD_600_ was recorded using a Spectramax i3 taking 200 µL of the dilution into a 96 well plate. 5 mL cultures were permeabilized by adding 30 µL of a 0.1% SDS solution and 30 µL of toluene. After vigorous mixing, the tubes were left in an incubator at 37°C for 40 minutes to allow for toluene evaporation. The enzymatic reaction was started by transferring 500 µL of permeabilized cells to a fresh glass tube containing 500 µL of PM2 buffer (70 mM Na_2_HPO_4_, 30 mM NaH_2_PO_4_, 1 mM MgSO_4_, 0.2 mM MnSO_4_, 100 mM β-mercaptoethanol, and 0.1% *O*-nitrophenol-β-d-galactoside (ONPG) pH 7.0) and allowing reaction to proceed at 28°C for 2 hr. Control wells were made by adding M63 media to the PM2. The reaction was stopped by addition of 0.5 M Na_2_CO_3_. OD_420_ was recorded and activity was measured by calculating:

A = 200 x (OD_420_ – OD_420_ in control tube) / min of incubation) x dilution factor (5)

One unit of β-galactosidase activity corresponds to 1 nmol of ONPG hydrolyzed/min at 28°C. The factor 200 is the inverse of the absorption coefficient of the o-nitrophenol, which is 0.005 per nmol/mL at pH 11.0 (after addition of Na_2_CO_3_). To account for differences in cell density, activity is reported in units/mg dry weight bacteria. OD600 was used as 1 mL of culture at OD_600_ = 1 corresponds to 300 µg dry weight bacteria (16, 17).


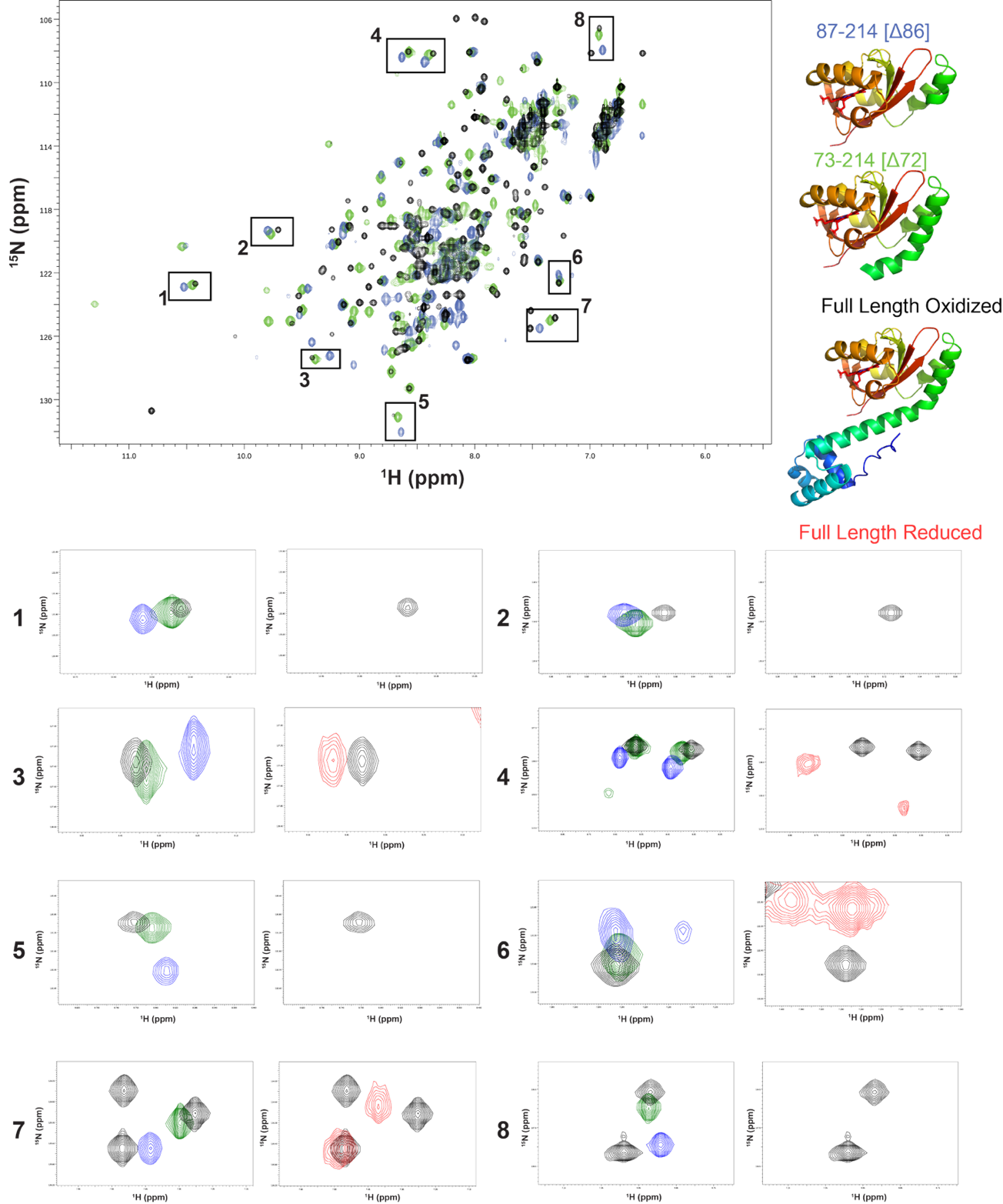


**Figure S1:** ^1^H/^15^N HSQC spectra of oxidized FG214 constructs: full length (Black), Δ72 (green), and Δ86 (blue). Boxes indicate peaks that display progressive perturbation in all constructs, and insets show regions compared to full length oxidized (black) and full length reduced (red).

**
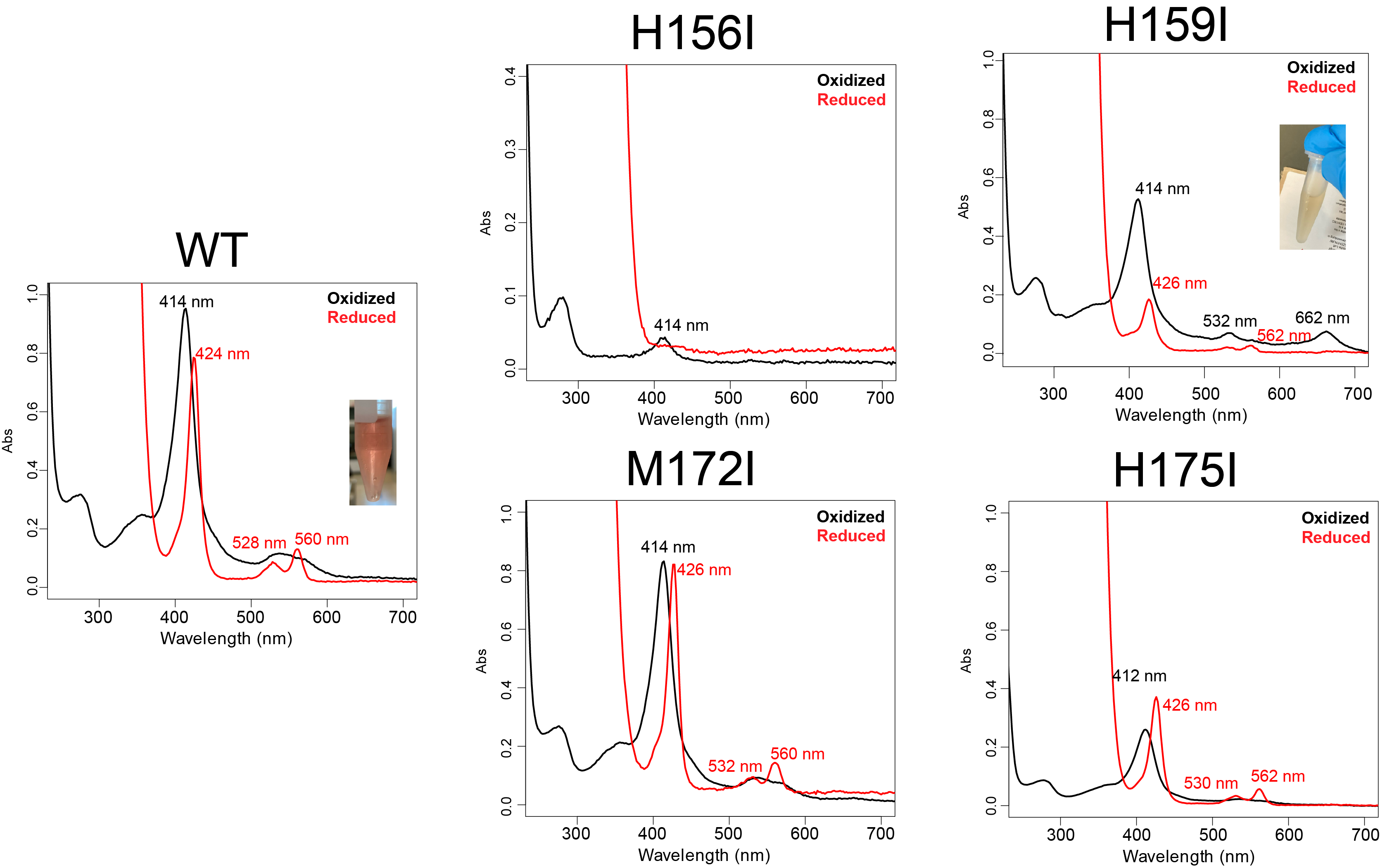
**

**Figure S2:** UV-visible absorbance spectra of FG214 wild-type (WT) and PAS domain point mutants under oxidized (black) and reduced (red) conditions. WT data are reproduced here from Figure 1d for ease of comparison.


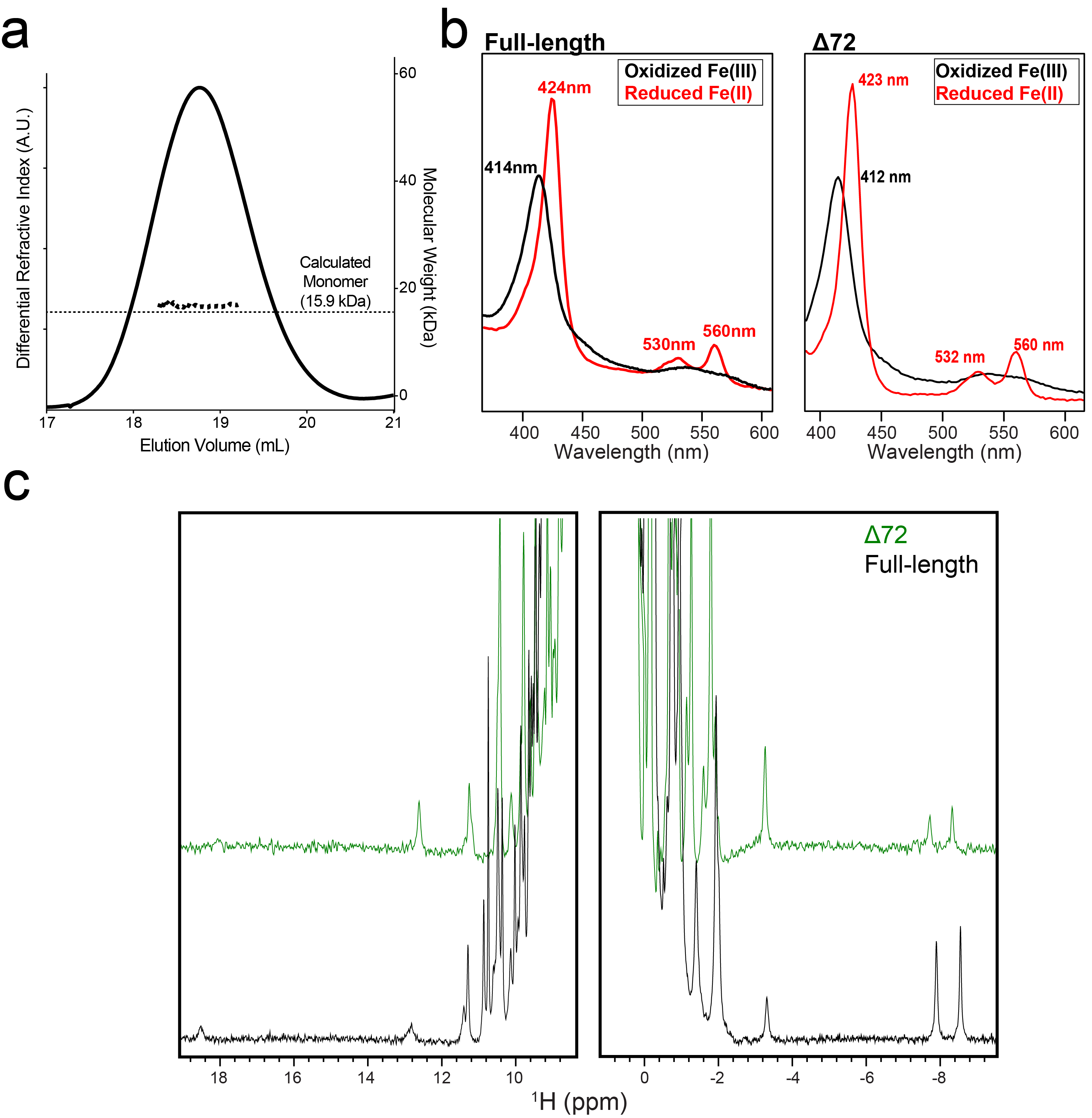


**Figure S3**: Solution characterization of FG214 (Δ72). **a)** SEC-MALS analysis of FG214 (Δ72) compared to calculated monomeric molecular weight. **b)** UV-visible absorbance spectra of oxidized (black) and reduced (red) FG214 (Δ72) compared to full-length data as shown in Figure 1. **c)** ^1^H NMR spectra of oxidized FG214 (Δ72) (green) compared to full-length (black, reproduced from Figure 1f).

**
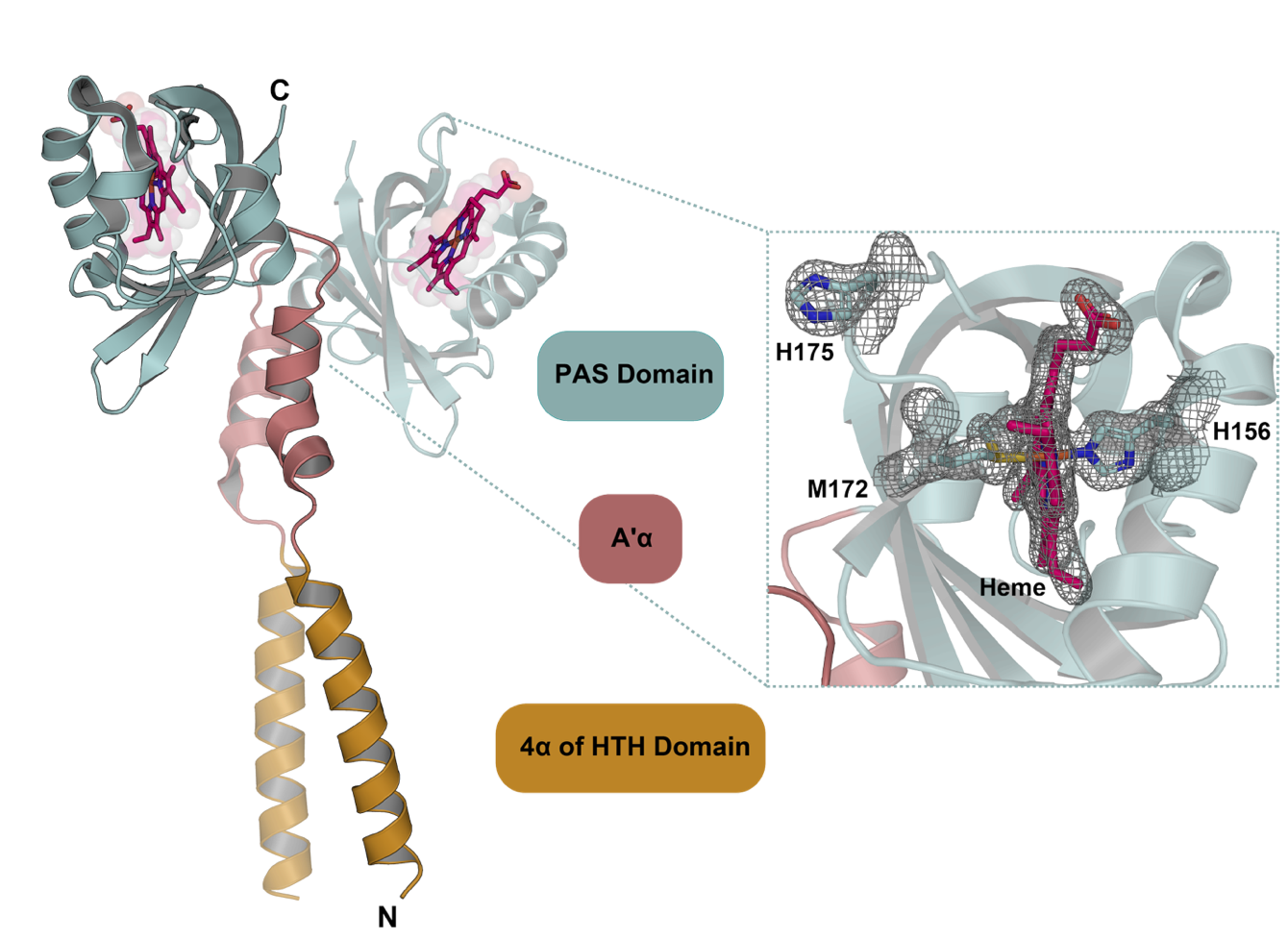
**

**Figure S4:** 1.65 Å crystal structure of FG214(Δ72) from the same crystal conditions yielding the structure from Fig. 4 but cryoprotected following addition of soluble sodium dithionite. PDBID: 10JY.

**
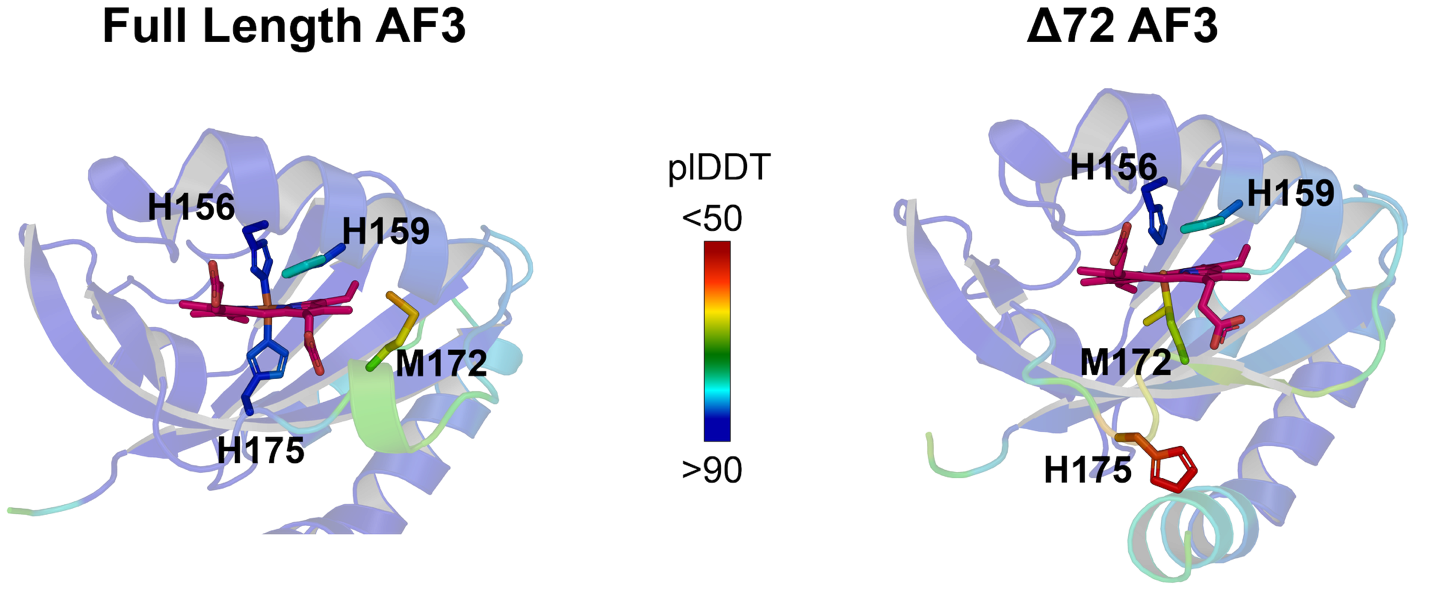
**

**Figure S5:** AlphaFold3 model of heme binding pocket predicted in full length model (left)

compared to Δ73 model (right).

**
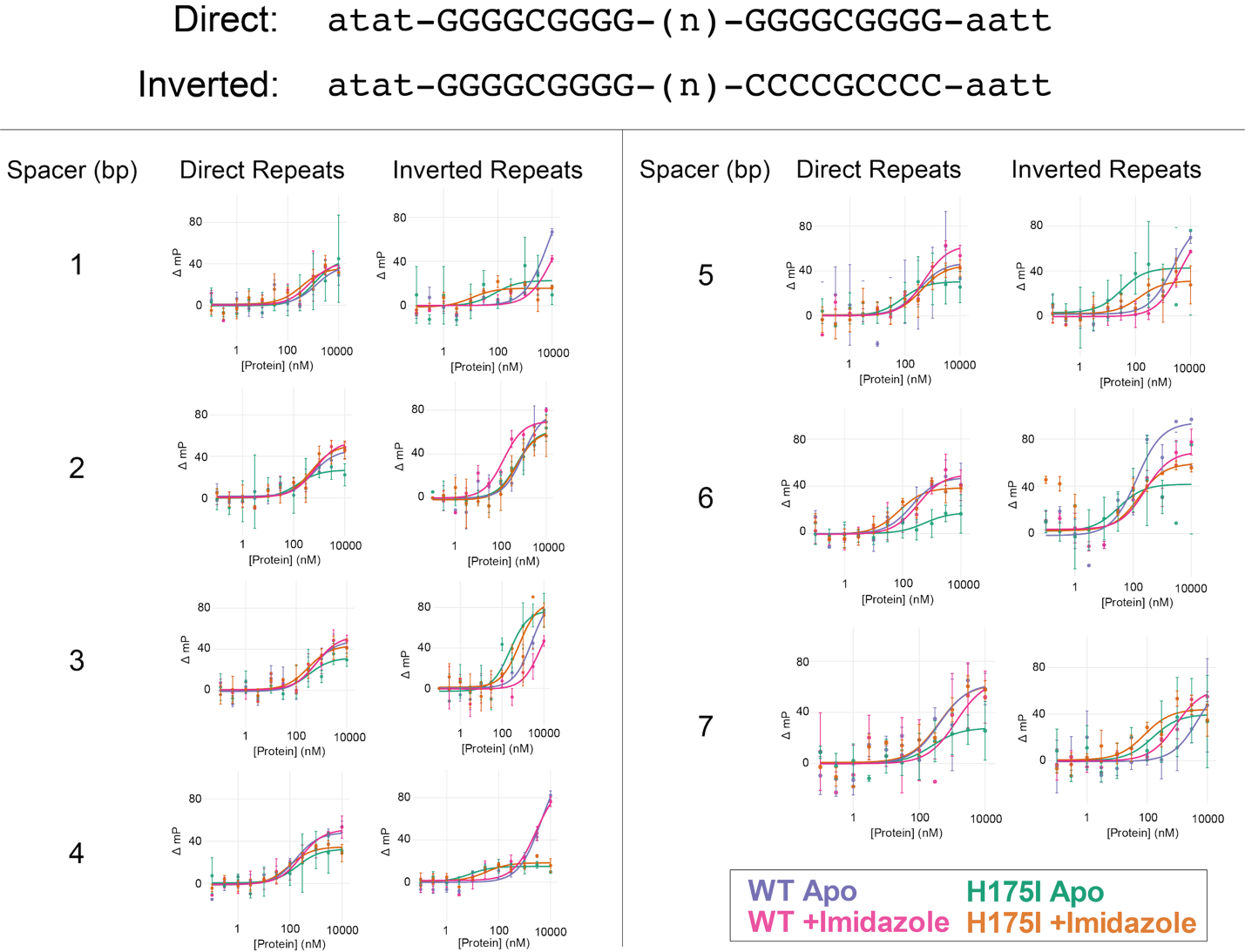
**

**Figure S6:** Fluorescence polarization analysis collected in triplicate for H175I apo (green), H175I + imidazole (orange), WT apo (purple), and WT + imidazole (pink).

**Table S1:** **UV-visible absorbance spectra peaks for FG214 constructs in oxidized and reduced conditions**

| **Mutant** | **State** | **Soret Band (nm)** | **Q Bands (nm)** |  | **Additional Peak (nm)** |
| --- | --- | --- | --- | --- | --- |
| WT | Oxidized | 414 | Broad 550 |  | Not observed |
|  | Reduced | 424 | 528 / 560 |  | Not observed |
| H156I | Oxidized | 414 | Not observed |  | Not observed |
|  | Reduced | Not observed | Not observed |  | Not observed |
| H159I | Oxidized | 414 | 532 |  | 662 |
|  | Reduced | 426 | 532 / 562 |  | Not observed |
| M172I | Oxidized | 414 | Broad 550 |  | Not observed |
|  | Reduced | 427 | 532 / 560 |  | Not observed |
| H175I | Oxidized | 412 | Broad 550 |  | Not observed |
|  | Reduced | 426 | 530 / 562 |  | Not observed |

| Table S2: FG214 Crystallographic Data Collection and Refinement Statistics | | |
| --- | --- | --- |
|  | FG214(78-219) | FG214(78-219)+DT Soak |
| Data Collection |  |  |
| Space Group | P2_1_ 2_1_ 2_1_ | P2_1_ 2_1_ 2_1_ |
| Unit cell parameters (Å) |  |  |
|  | a = 63.35 | a = 63.03 |
|  | b = 63.93 | b = 65.21 |
|  | c = 105.97 | c = 105.87 |
| Wavelength (Å) | 0.92019 | 0.97860 |
| Resolution Range (Å) | 34.451 – 1.467 (1.493 – 1.467) | 55.523 – 1.648 (1.677 – 1.648) |
| Total reflections  Unique reflections | 583436  75338 (3680) | 393593  53371 (2645) |
| Multiplicity | 7.7 (7.0) | 7.4 (7.7) |
| Completeness (%) | 99.9 (99.9) | 100.0 (99.9) |
| Mean *I*/σ(*I*) | 9.8 (1.5) | 11.0 (0.6) |
| R_meas_ | 0.115 (2.397) | 0.093 (3.327) |
| CC_1/2_ | 0.992 (0.335) | 0.999 (0.382) |
|  |  |  |
| Refinement |  |  |
| Reflection used in refinement | 75301 (2823) | 53262 (2716) |
| Reflections used in R-free | 3708 (132) | 2634 (141) |
| Number of non-hydrogen atom | 2656 | 2563 |
| Macromolecules | 2333 | 2286 |
| Ligands | 139 | 105 |
| Solvent | 183 | 172 |
| R_work_ | 0.1641 (0.3044) | 0.1899 (0.3734) |
| R_free_ | 0.1901 (0.2984) | 0.2150 (0.3636) |
| RMS(bonds) | 0.006 | 0.008 |
| RMS(angles) | 0.87 | 0.93 |
| Protein residues | 282 | 286 |
| Average B-factor | 29.1 | 38.67 |
| Macromolecules | 27.99 | 38.58 |
| Ligands | 37.05 | 34.5 |
| Solvent | 36.76 | 42.5 |
| Ramachandran plots |  |  |
| Favored (%) | 98.55 | 100.0 |
| Allowed (%) | 1.09 | 0.00 |
| Outliers (%) | 0.36 | 0.00 |
|  |  |  |
| PDB accession code | 10JX | 10JY |
| Statistics for the highest-resolution shell are shown in parentheses. | | |

**Table S3: DNA Sequences used in Figure 5C**

| **Name** | **Sequence (5ʹ-3ʹ)** |
| --- | --- |
| **IR2** | ATATGGGGCGGGGATCCCCGCCCCAATT |
| **DR2** | ATATGGGGCGGGGATGGGGCGGGGAATT |

**Supporting References**

1. S. M. Harper, L. C. Neil, K. H. Gardner, Structural Basis of a Phototropin Light Switch. *Science* **301**, 1541-1544 (2003).

2. R. B. Kapust *et al.*, Tobacco etch virus protease: mechanism of autolysis and rational design of stable mutants with wild-type catalytic proficiency. *Protein Engineering, Design and Selection* **14**, 993-1000 (2001).

3. J. Phan *et al.*, Structural Basis for the Substrate Specificity of Tobacco Etch Virus Protease. *Journal of Biological Chemistry* **277**, 50564-50572 (2002).

4. V. S. Manu, C. Olivieri, K. Pavuluri, G. Veglia, Design and applications of water irradiation devoid RF pulses for ultra-high field biomolecular NMR spectroscopy. *Physical Chemistry Chemical Physics* **24**, 18477-18481 (2022).

5. M. Norris, B. Fetler, J. Marchant, B. A. Johnson, NMRFx Processor: a cross-platform NMR data processing program. *Journal of Biomolecular NMR* **65**, 205-216 (2016).

6. E. Koag *et al.* (2025) NMRFx: Integrated Software for NMR Data Processing, Visualization, Analysis and Structure Calculation. (Cold Spring Harbor Laboratory).

7. C. Lu, M. L. Wells, A. Reckers, S. K. McBride, A. Glasgow, Site-resolved energetic information from HX–MS experiments. *Nature Chemical Biology* 10.1038/s41589-025-02049-1 (2025).

8. S. Stoll, A. Schweiger, EasySpin, a comprehensive software package for spectral simulation and analysis in EPR. *Journal of Magnetic Resonance* **178**, 42-55 (2006).

9. C. Vonrhein *et al.*, Data processing and analysis with the<i>autoPROC</i>toolbox. *Acta Crystallographica Section D Biological Crystallography* **67**, 293-302 (2011).

10. J. Agirre *et al.*, The <i>CCP</i>4 suite: integrative software for macromolecular crystallography. *Acta Crystallographica Section D Structural Biology* **79**, 449-461 (2023).

11. P. Emsley, B. Lohkamp, W. G. Scott, K. Cowtan, Features and development of <i>Coot</i>. *Acta Crystallographica Section D Biological Crystallography* **66**, 486-501 (2010).

12. D. Liebschner *et al.*, Macromolecular structure determination using X-rays, neutrons and electrons: recent developments in <i>Phenix</i>. *Acta Crystallographica Section D Structural Biology* **75**, 861-877 (2019).

13. M. F. Berger *et al.*, Compact, universal DNA microarrays to comprehensively determine transcription-factor binding site specificities. *Nature Biotechnology* **24**, 1429-1435 (2006).

14. M. F. Berger, M. L. Bulyk, Universal protein-binding microarrays for the comprehensive characterization of the DNA-binding specificities of transcription factors. *Nature Protocols* **4**, 393-411 (2009).

15. M. G. Olson, M. Goldammer, E. Gauliard, D. Ladant, S. P. Ouellette, "A Bacterial Adenylate Cyclase-Based Two-Hybrid System Compatible with Gateway® Cloning". (Springer New York, 2018), 10.1007/978-1-4939-7871-7_6, pp. 75-96.

16. G. Karimova, N. Dautin, D. Ladant, Interaction Network among<i>Escherichia coli</i>Membrane Proteins Involved in Cell Division as Revealed by Bacterial Two-Hybrid Analysis. *Journal of Bacteriology* **187**, 2233-2243 (2005).

17. A. Hosseini, J. Mas, The β-galactosidase assay in perspective: Critical thoughts for biosensor development. *Analytical Biochemistry* **635**, 114446 (2021).
